# Eating Behaviours of Preadolescent Children over Time: Stability, Continuity and the Moderating Role of Perceived Parental Feeding Practices

**DOI:** 10.3390/ijerph13040437

**Published:** 2016-04-20

**Authors:** Laura Houldcroft, Claire Farrow, Emma Haycraft

**Affiliations:** 1 School of Sport, Exercise and Health Sciences, Loughborough University, Loughborough, Leicestershire LE11 3TU, UK; l.a.houldcroft@lboro.ac.uk (L.H.); e.haycraft@lboro.ac.uk (E.H.); 2 Department of Psychology, School of Life and Health Sciences, Aston University, Birmingham, West Midlands B4 7ET, UK

**Keywords:** eating behaviours, preadolescent, parent, feeding practices, pressure, restriction, dietary restriction, emotional eating, external eating, stability, continuity

## Abstract

The links between childhood eating behaviours and parental feeding practices are well-established in younger children, but there is a lack of research examining these variables in a preadolescent age group, particularly from the child’s perspective, and longitudinally. This study firstly aimed to examine the continuity and stability of preadolescent perceptions of their parents’ controlling feeding practices (pressure to eat and restriction) over a 12 month period. The second aim was to explore if perceptions of parental feeding practices moderated the relationship between preadolescents’ eating behaviours longitudinally. Two hundred and twenty nine preadolescents (mean age at recruitment 8.73 years) completed questionnaires assessing their eating behaviours and their perceptions of parental feeding practices at two time points, 12 months apart (T1 and T2). Preadolescents’ perceptions of their parental feeding practices remained stable. Perceptions of restriction and pressure to eat were continuous. Perceptions of parental pressure to eat and restriction significantly moderated the relationships between eating behaviours at T1 and T2. The findings from this study suggest that in a preadolescent population, perceptions of parental pressure to eat and restriction of food may exacerbate the development of problematic eating behaviours.

## 1. Introduction

Parents are responsible for the majority of feeding interactions that their children experience and parental control over children’s food intake and, in particular, the predictors of such control have been researched extensively [1,2,3]. Although younger children have less autonomy in relation to their food choices and eating environment [4], parental influences over food and eating continue to be relevant throughout childhood [5,6,7] and parents report using controlling feeding practices with young children through to adolescents [8,9,10,11,12]. High levels of general control over children’s feeding have been shown to be counterproductive [1,9] and have been linked with children’s inability to respond appropriately to internal hunger and satiety signals, as they instead associate the process of eating with external cues [12,13], or with disordered eating behaviours [11].

The two most commonly studied controlling feeding practices are *pressure to eat* and *restriction* [14]. Parental pressure for their child to eat refers to parents’ tendency to pressure their child to eat more, typically occurring during mealtimes [14]. Pressuring children to eat foods that are healthy or their parents perceive as being good for them, such as fruit and vegetables, is a commonly reported practice with young children [14] and has been linked to lower fruit and vegetable intake and picky eating longitudinally [15], as well as reduced weight gain longitudinally [3]. Although research has found associations between mothers’ reported use of pressure to eat when and their perceptions that their child is underweight [16], experimental research suggests that the good intentions of pressure can often have counterproductive outcomes, such as children actually eating *less* of a pressured food [17].

A second commonly used controlling feeding practice is restriction of food, whereby parents overtly or covertly restrict, or limit, the type or amount of food that their child eats, typically at mealtimes or with snacks [14]. Restriction usually relates to limiting access to unhealthy foods but can also be applied to reducing food intake more generally [14]. In children aged 7–11 years, perceived parental restriction has been shown to be the type of food management most frequently reported by children [18]. Experimental and longitudinal research has shown that restriction *increases* a child’s intake of restricted foods and promotes general over-eating [19], with children with higher appetites and lower inhibitory control at increased susceptibility to the effects of parental restriction [20].

To date, limited research has examined perceptions of parental controlling feeding practices and the associations with problematic eating. Cross-sectional studies with younger girls [13] and an adolescent population [11] have found that perceptions of greater controlling parental feeding practices are associated with reports of greater unhealthy eating behaviours. In two cross-sectional studies using preadolescents, similar results have been reported. Van Strein and Bazelier found that perceptions of higher levels of parental pressure to eat were associated with greater reports of emotional and external over-eating in boys (but not girls) [21]. A UK study reported similar results, with higher perceived levels of parental pressure to eat related to higher self-reported levels of dietary restraint, external eating and emotional eating (for both genders) [22]. Both studies reported that perceptions of parental restriction were negatively related to preadolescents’ emotional and external eating, although van Strien and Bazelier [21] also found a positive association between restriction and restrained eating behaviours. These findings highlight the important associations between perceptions of controlling parental feeding practices and child obesogenic and under-eating behaviours in preadolescence, and further research extending these cross-sectional findings is warranted.

Previous research in the parent-child feeding domain has often focussed on parents’ self-reported use of controlling feeding practices with their young children [2]. It is possible that parental reports and child perceptions may differ. Carper and colleagues [13] compared mothers’ self-reported controlling feeding practices with child perceptions of their mothers’ use of feeding practices in girls aged 5 years and reported positive associations in reports of pressure to eat, but not restriction. A more recent study with preadolescents found that perceptions of parental pressure and restriction were correlated with parental reports of such behaviours for older children (10.45 years), although not for younger children (8.21 years) [23]. Given the mixed results, the associations between child perceptions of controlling parental feeding practices and child maladaptive eating behaviours warrant further investigation, particularly during the somewhat under-researched preadolescent period.

A further area of limited research in the preadolescent age group is the continuity and stability of their perceptions of controlling parental feeding practices. Continuity and stability are two distinct concepts that developmentalists have used to explore the development of behaviours over time. Continuity of a behaviour refers to the consistency in the mean level of a behaviour, e.g., eating behaviours, over time. A continuous eating behaviour is one where the mean level of the eating behaviour is the same at one time point as a second, later time point. A discontinuous behaviour is one where the mean level of the eating behaviour changes over time. Stability refers to the consistency in ranks of behaviour over time. For example, a stable eating behaviour would be one where some children report high levels at one point in time and again at a later time point, and other children report low levels at both time points. An unstable behaviour occurs when rank order is not maintained [24,25]. In younger children, the use of controlling feeding practices reported by parents has been shown to be stable [1,25]. In relation to continuity, parents report using less pressure to eat and restriction with their children between 7 and 10 years of age [26]. However, to date, there has been no research that has looked at both the continuity and stability of parental feeding practices across a preadolescent time period, or from the perspective of the child rather than the parent.

The present study aimed to examine the continuity and stability of preadolescent reports of their parents’ controlling feeding practices (pressure to eat and restriction) over a 12 month period. It was anticipated, based on previous research conducted with parents [26], that preadolescents’ perceptions of their parents’ use of controlling feeding practices would be discontinuous, decreasing over time as children assert more autonomy over eating as they age. It was further anticipated, based on research with younger children [1,25], that perceptions of parental controlling feeding practices would be stable over a 12 month period. Further, based on the longitudinal links between controlling parental feeding practices and child maladaptive eating behaviours [3,15,19,20], and to extend the findings of previous research with a preadolescent population [21,22], this study aimed to explore how perceptions of controlling feeding practices may moderate the relationship between preadolescents’ eating behaviours over time.

## 2. Experimental Section

### 2.1. Participants

At baseline (T1), 343 children from eight UK primary schools took part in the study. At 12 month follow-up (T2), six schools and 254 of the original sample (71%) remained in the study, with 229 having complete data required for the present study. The two schools cited a change in Head Teacher and conflicting time commitments as their reasons for not participating in the follow-up. Some individual children from T1 were absent on the day of T2 collection or failed to complete the full questionnaire measures. However, no children actively opted out, or were opted out of the research by their parents, at T2. The data reported in this paper concern the 229 children who provided full data at both time points (T1 and T2).

The final sample consisted of a roughly equal number of boys and girls (boys *n* = 120, girls *n* = 109. At T1 the participants ranged in age from 7.25 to 10.25 years (M = 8.73 years, Standard Deviation (SD) = 0.57), and at T2 from 8.25 to 11.25 years (M = 9.73 years, SD = 0.62). The majority (94%) of the final T2 sample reported their ethnicity as White British.

### 2.2. Procedure and Measures

Primary schools at T1 were recruited via letters and telephone calls. These schools were again contacted at T2 and of the eight schools that participated at T1, six remained in the study at T2. Participating schools sent letters to parents of children in the relevant aged classes outlining the study, and allowing them to opt out their child from participating. The research was approved by the University Ethics Approvals (Human Participants) Sub-Committee (ethical approval number: G07-P4). The study was conducted as part of a class lesson and children verbally assented to participate. They completed a questionnaire pack at T1 and T2, consisting of the measures outlined below.

#### 2.2.1. Eating Patten Inventory for Children (EPIC)

To measure children’s self-reported eating behaviours, three subscales of the EPIC [27] were used: dietary restraint (e.g., “To keep my weight, I often eat less than I would actually like to”; eight items); external eating (e.g., “When I see someone eat, I also get hungry”; five items); and emotional eating (e.g., “Eating helps me when I am disappointed”; four items), totalling 17 question items. Children responded on a four point scale ranging from “not at all” (1) to “totally” (4). Higher scores on the EPIC subscale signify a higher level of maladaptive eating behaviour. The EPIC has adequate factor structure [27] and previous research suggests good validity when used with preadolescents [27,28]. The internal reliability coefficients (Cronbach’s α) were: dietary restraint α 0.85 (T1), α 0.88 (T2); external eating α 0.78 (T1) α 0.82 (T2); and emotional eating α 0.75 (T1) α 0.79 (T2); all demonstrating acceptable levels of internal consistency.

#### 2.2.2. Kids’ Child Feeding Questionnaire (KCFQ)

To measure children’s perceptions of the feeding practices used by their parents, the KCFQ [12] subscales of pressure to eat (e.g., “When you say “I’m not hungry” at dinnertime, do your parents say “You need to eat anyway”?”; eight items) and restriction (e.g., “Do your parents ever say things like “You’ve had enough to eat now, you need to stop”?”; five items) were used, totalling 13 items. The KCFQ is the only known measure of children’s perceptions of parental feeding practices. The authors of the KCFQ suggest that questions be administered twice, measuring each parent’s behaviours separately (e.g., “Does your mommy ever let you have snacks?” and “Does your daddy ever let you have snacks?”). In the present study, however, these were combined and questions were asked once by replacing “mommy/daddy” with “parents” in order to minimise child fatigue in the young age sample, and to replicate the use of the measure by previous research [21,22]. To allow comparisons to be made with previous work, the factor structure of the KCFQ adopted by earlier studies [21,22] was likewise used in the current study. The KCFQ has a three point response scale; “no”, “sometimes” and “yes”, with higher scores suggesting greater perceived levels of parental controlling feeding practices. Internal reliability coefficients (Cronbach’s α) suggested adequate levels of internal reliability: pressure to eat α 0.68 (T1), α 0.56 (T2); and restriction α 0.60 (T1) α 0.62 (T2). These values are broadly in line with those obtained when the measure was developed [12].

### 2.3. Statistical Analyses

Histograms, skew and kurtosis data for each subscale indicated that the sample did not deviate substantially from normality and parametric tests were used in all analyses. The sample size of 229 exceeded the requirements to detect a medium effect at *p* < 0.05 with a power of 0.80 when using correlations (*n* = 85), tests of difference (*n* = 64) and moderated regression (*n* = 177) [29]. There were no significant differences between children who remained in the study at T2 compared to those who were only involved at baseline in terms of age, eating behaviours or perceptions of parental controlling feeding practices (*p* > 0.05; data not shown).

To examine the continuity of perceived parental feeding practices over time, mean difference scores were calculated between T1 and T2 reports of these behaviours. Paired samples t-tests were conducted to calculate continuity. Positive mean scores indicated an average increase in perception of the feeding practice over 12 months, whereas a negative mean score indicated an average decrease. To examine the stability of perceived parental feeding practices over time, two-tailed Pearson’s correlations were calculated. Positive correlations indicated stability in perceptions of parental feeding practices.

Moderated regression analyses [30] were used to explore whether perceived parental feeding practices (pressure to eat and restriction) moderated the relationship between preadolescents’ own reports of eating behaviours at T1 and T2. Moderation analyses were tested by calculating the main and interaction effects of perceived parental feeding practices at T1 and T2 in predicting preadolescents’ reported eating behaviours at T1 and T2. Simple slope analyses were used to explore any significant moderations. All tests were two-tailed due to the exploratory nature of the hypotheses and the *p*-value was set at <0.05.

## 3. Results and Discussion

### 3.1. Descriptive Statistics and Gender Differences

Table 1 displays the mean, standard deviation *(SD)*, and test of difference scores for preadolescents’ individual eating behaviours by gender at baseline (T1) and follow-up (T2).

Mean scores for the EPIC eating behaviours at T2 were comparable to previous research with children of this age range [27,28]. Independent *t*-tests of difference showed no gender differences in reports of eating behaviours for boys and girls, differing to previous research with preadolescents which found that boys reported greater levels of external eating [20,21] and emotional eating [20]. Two-tailed Pearson’s correlations indicated that child age was negatively associated with external eating at T1 (*r* = −0.219, *p* = 0.001) and T2 (*r* = −0.198, *p* = 0.003); emotional eating at T1 (*r* = −0.304, *p* = 0.000) and T2 (*r* = −0.246, *p* = 0.000). Age was not associated with reports of dietary restraint behaviours. Subsequent analyses using external eating and emotional eating controlled for age, where applicable.

Table 2 displays the mean, standard deviation (SD) and test of difference scores (*t*) for preadolescents’ perceptions of their parents’ feeding practices by gender at T1 and T2. Mean scores for the KCFQ were comparable to the results reported with the original questionnaire [15]. Independent samples *t*-tests of difference (Table 2) showed no significant differences between boys’ and girls’ perceptions of parental feeding practices at T1 and T2. 

Two-tailed Pearson’s correlations indicated that child age was not associated with perceived parental feeding practices at either T1 or T2. Independent *t*-tests of difference showed no significant differences in reports of eating behaviours, perceived parental feeding practices or age, for children who remained in the study at T2 compared to those who did not.

### 3.2. Stability of Perceived Parental Feeding Practices between T1 and T2

To examine the stability of children’s perceptions of their parents’ feeding practices over time, a series of two-tailed Pearson’s correlations were calculated (Table 3). As Table 3 shows, both perceptions of pressure to eat and perceptions of restriction were significantly positively correlated over time, suggesting good stability in perceptions of parental feeding practices in preadolescent children.

### 3.3. Continuity of Perceived Parental Feeding Practices over 12 Months

To examine the continuity of children’s perceptions of their parents’ use of controlling feeding practices over time, mean change scores for variables between T1 and T2 were initially calculated, followed by paired samples *t*-tests (Table 4). Table 4 shows that preadolescents’ perceptions of parental restriction and pressure to eat were continuous between T1 and T2.

### 3.4. Moderation Analyses

Moderated regression analyses [30] were used to examine under what conditions of a moderating variable preadolescents’ eating behaviours were stable over time. Moderated regressions were used to explore if the relationships between eating behaviours at T1 and T2 were moderated by perceptions of controlling feeding practices at T1 and T2. Moderated regressions that used external eating (T1 and T2) and emotional eating (T1 and T2) as variables controlled for age in Step 1 (as age was previously found to correlate with these variables). Simple slope analyses were used to further investigate any significant moderation pathways found. Simple slopes for the regression of T1 eating behaviour on T2 eating behaviour were computed at three levels of the moderator: low (−1 Standard Deviation (SD) below the mean), moderate (mean) and high (+1 SD above the mean).

#### 3.4.1. The Moderating Role of Perceived Pressure to Eat on the Stability of Eating Behaviours between T1 and T2

The interactions between the over-eating behaviour, emotional eating and perceptions of parental pressure to eat at T1 (*β* = −0.102, *SE* = 0.139, *p* = 0.463) and T2 (*β* = −0.218, *SE* = 0.167, *p* = 0.192), failed to significantly predict emotional eating behaviours at T2. Similarly, the interactions between the over-eating behaviour, external eating and perceptions of pressure to eat at T1 (*β* = −0.186, *SE* = 0.131, *p* = 0.156) and T2 (*β* = −0.128, *SE* = 0.136, *p* = 0.349), failed to significantly predict external eating behaviours at T2. Further, the interaction between dietary restraint (T1) and perceptions of parental pressure to eat at T1 failed to significantly predict dietary restraint behaviours at T2 (*β* = −0.025, *SE* = 0.140, *p* = 0.855).

##### Pressure to eat at T2 as a Moderator of Dietary Restraint between T1 and T2

**Figure 1:**
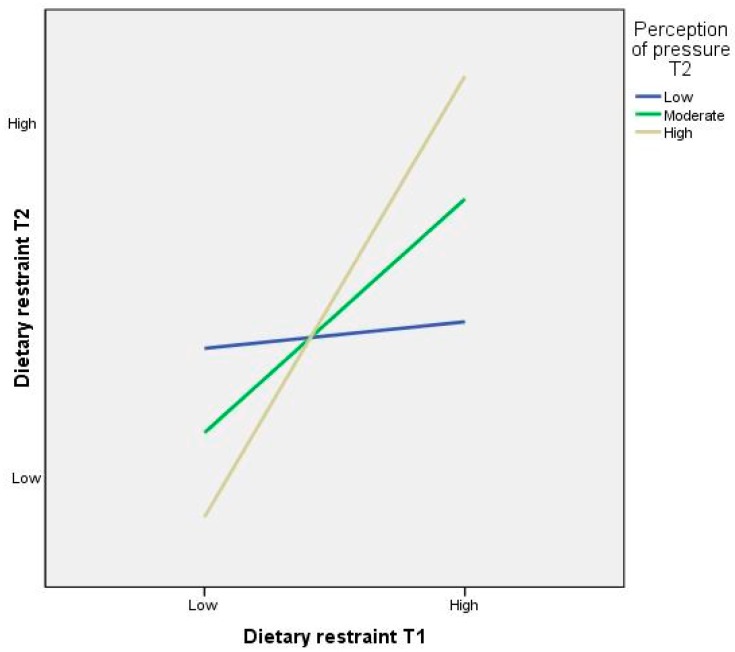
Simple regression slopes for the moderating role of preadolescents’ perceptions of parental pressure to eat (time point 2) on the relationship between time point 1 (Mean age = 8.73 years) and time point 2 (Mean age = 9.73 years) dietary restraint behaviours.

The interaction between dietary restraint behaviours at T1 and perceptions of parental pressure to eat at T2 was a significant predictor of dietary restraint behaviours at T2 (*β* = 0.363, *SE* = 0.168, *p* = 0.032). The interaction between dietary restraint and perceptions of pressure to eat was significant when the moderator was high, moderate or low. The relationship was stronger when perceived levels of parental pressure were high (*β* = 1.413 *SE* = 0.016, *p* = 0.000) than when they were moderate (*β* = 0.786, *SE* = 0.011, *p* = 0.000) or low (*β* = 0.158, *SE* = 0.015, *p* = 0.000). Figure 1 shows the simple slopes plots for the interaction.

#### 3.4.2. The Moderating Role of Perceived Restriction on the Stability of Eating Behaviours between T1 and T2

The interactions between dietary restraint at T1, and perceptions of restriction at T1 (*β* = 0.230, *SE* = 0.121, *p* = 0.060) and T2 (*β* = 0.086, *SE* = 0.145, *p* = 0.554), failed to significantly predict dieting behaviours at T2. Likewise, the interactions between external eating behaviours at T1 and perceptions of restriction at T1 (*β* = −0.162, *SE* = 0.107, *p* = 0.132) and T2 (*β* = −0.083, *SE* = 0.107, *p* = 0.440), failed to significantly predict external eating behaviours at T2. The interactions between emotional eating behaviours (T1) and perceptions of parental restriction at both T1 *and* T2 were significant predictors of emotional eating behaviours at T2.

##### Restriction at T1 as a Moderator of Emotional Eating between T1 and T2

**Figure 2:**
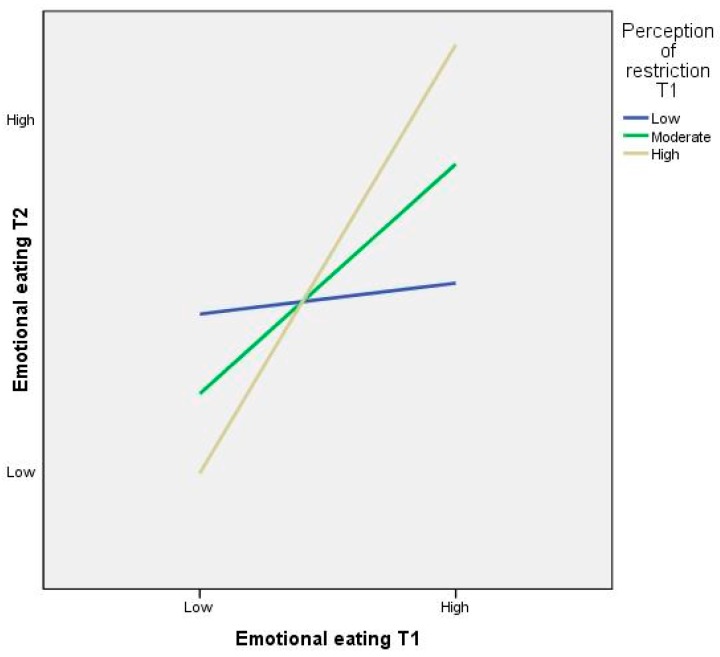
Simple regression slopes for the moderating role of preadolescents’ perceptions of parental restriction (time point 1) on the relationship between time point 1 (Mean age = 8.73 years) and time point 2 (Mean age = 9.73 years) emotional eating behaviours.

The interaction between emotional eating behaviours at T1 and perceptions of parental restriction at T1 was a significant predictor of emotional eating behaviours at T2 (*β* = −0.249, *SE* = 0.117, *p* = 0.034). The interaction between emotional eating and perceptions of restriction at T1 was significant at all levels of the moderator. The relationship was stronger when perceived levels of restriction were high (*β* = 1.419, *SE* = 0.018, *p* = 0.000) than when they were moderate (*β* = 0.761, *SE* = 0.012, *p* = 0.000), or low (*β* = 0.102, *SE* = 0.018, *p* = 0.000). Figure 2 shows the simple slopes plots for the interaction.

##### Restriction at T2 as a Moderator of Emotional Eating between T1 and T2

**Figure 3:**
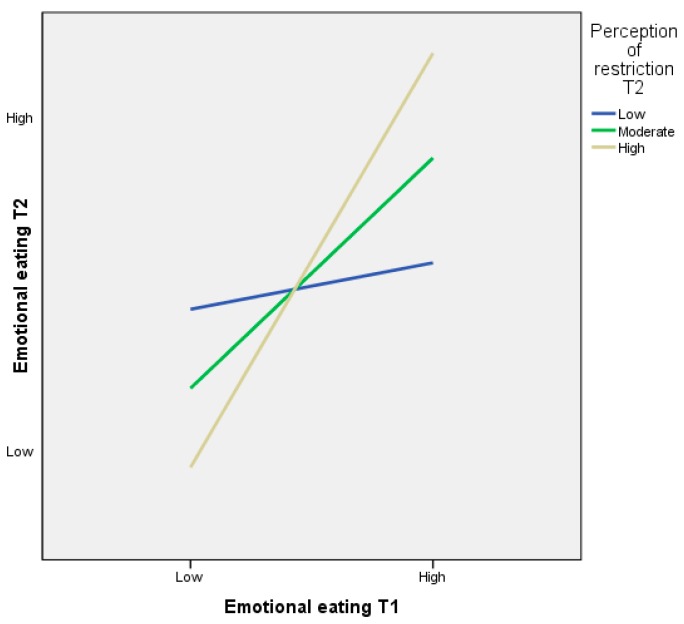
Simple regression slopes for the moderating role of preadolescents’ perceptions of parental restriction (time point 2) on the relationship between time point 1 (Mean age = 8.73 years) and time point 2 (Mean age = 9.73 years) emotional eating behaviours.

The interaction between emotional eating behaviours at T1 and perceptions of parental restriction at T2 was a significant predictor of emotional eating behaviours at T2 (*β* = −0.378, *SE* = 0.120, *p* = 0.002). The interaction between emotional eating and perceptions of restriction at T2 was significant at all levels of the moderator. The relationship was stronger when perceived levels of restriction were high (*β* = 1.485, *SE* = 0.016, *p* = 0.000) than when they were moderate (*β* = 0.787, *SE* = 0.010, *p* = 0.000), or low (*β* = 0.089, *SE* = 0.014, *p* = 0.000). Figure 3 shows the simple slopes plots for the interaction.

### 3.5. Discussion of Study Findings

The current study aimed to examine the continuity and stability of preadolescents’ perceptions of their parents’ controlling feeding practices, namely pressure to eat and restriction, over a 12 month period. Contrary to the hypothesised findings, perceptions of parental pressure to eat and restriction were continuous over time. As anticipated, perceptions of pressure to eat and restriction remained stable over time.

The second aim, to explore if perceptions of parental feeding practices moderated the relationship between preadolescents’ eating behaviours at T1 and T2, produced mixed results. Preadolescents’ perceptions of pressure to eat moderated the relationship between under-eating behaviour (dietary restraint) at T1 and T2 (although this was only found for perceptions of pressure at T2 and not T1). Perceptions of restriction at T1 and T2 moderated the relationship between emotional eating at T1 and T2. However, perceptions of restriction at T1 and T2 did not moderate the relationship between external eating at T1 and T2.

The finding that perceptions of pressure to eat and restriction were continuous and stable over time is in contrast to previous research conducted with parents, who have reported using less pressure to eat and restriction with children aged 7 to 10 years of age, compared to when their children were younger [26]. Although children become more autonomous over their own eating with age and may rely less on parental influences on their intake [6], the preadolescent sample reported on here (with a mean age of 9.73 years at T2) did not perceive a reduction in their parents’ use of controlling feeding practices. The present study was based on *child* perceptions rather than parental reports of controlling feeding practices with their children, which could explain the differences in the results found. Results are limited by the use of child perceptions of their parents’ behaviours, in particular for restriction, as restriction of a child’s food is often a covert behaviour that may be unobservable by the child [31].

Perceptions of pressure to eat at T2 were found to moderate the relationship between dietary restraint at T1 and T2. This relationship was evident for high, moderate and low levels of pressure to eat, although strongest for higher perceived levels of pressure to eat, and weakest for lower perceived levels of parental pressure to eat. This suggests that when perceptions of parental pressure to eat are high, dietary restraint behaviours in preadolescents show greater consistency over time. This finding could suggest that parents may use pressure to eat with their children who exhibit dietary restraint behaviours in an attempt to increase their dietary intake, but this could be counterproductive. Furthermore, although a weaker relationship was found for preadolescents reporting lower perceived levels of parental pressure to eat, even lower levels of pressure to eat were associated with preadolescents’ continuing dietary restraint behaviours. This aligns with research with younger children which finds links between pressuring a child to eat and reduced intake of pressured food, and less weight gain [3,17].

Preadolescents’ perceptions of parental restriction at both T1 and T2 significantly moderated the relationship between emotional eating at T1 and T2. The relationships between emotional eating at T1 and T2 were significant for children who reported high, moderate and low levels of restriction. The relationship between emotional eating at T1 and T2 was strongest for children exposed to the greatest levels of restriction, suggesting that when preadolescents perceive higher parental restriction over their eating, they report more consistency in their emotional eating behaviours. Previous literature suggests that parents may pressure their child to eat more in an attempt to increase the intake of children who they are concerned about displaying dietary restraint behaviours [16]. In contrast, parents may restrict the intake of a child they believe to be exhibiting over-eating behaviours, such as emotional over-eating [12]. These parental strategies may be counterproductive and instead serve to maintain consistency in these maladaptive eating behaviours over time [32].

This study extends previous research by employing a longitudinal design to explore preadolescents’ eating behaviours and perceptions of parental controlling feeding practices. Both a strength and a limitation of the present study is the reliance on preadolescents’ perceptions of their parents’ feeding practices. Whilst using preadolescents’ reports gives us valuable insight into how they perceive their parents’ feeding practices, it is also possible that their perceptions may not be entirely accurate, particularly for restriction which suffered from a low reliability at T2, which might prevent wholly accurate identification of changes over time. Restriction can be conducted more covertly (e.g., by not keeping a particular food in the house) than pressure to eat, and so may not always be detectable to children [31]. Future research would benefit from collecting data from parents and linking this to children’s reports of controlling feeding practices. It would also be of interest to examine if children are better able to perceive controlling feeding practices accurately as they become older. A further limitation is that the results found both T1 and T2 variables moderated the stability of eating behaviours over time, specifically for emotional eating and perceived levels of restriction. T1 variables were examined as moderators but no significant results were found for dietary restraint and external eating. This could suggest a possible shift occurring between T1 and T2 in terms of the impact of such feeding practices and eating behaviours which in itself is an interesting finding; one which lends support to the notion that the period around adolescence is a time of significant change [33].

## 4. Conclusions

The present study is the first to explore whether perceived parental controlling feeding practices predict eating behaviours longitudinally in a preadolescent sample of boys and girls. No research to date has considered the continuity, stability and moderating role of perceived parental feeding practices across a preadolescent time period. The present findings therefore add to the existing literature by suggesting that perceived parental pressure to eat may have adverse effects on the development of dietary restraint behaviours, and perceived parental restriction may have negative effects on the development of emotional eating behaviours, in preadolescent children. These results require dissemination to health professionals working with families, and the parents of children in this age range, to educate them about the impact of their child’s perceptions of overly controlling feeding practices and to develop other more healthful feeding practices that support healthy eating in children.

## Figures and Tables

**Table 1:** Descriptive statistics (means, standard deviations) and tests of difference scores (*t*) for eating behaviours for boys and girls.

Variable	Mean (SD) Boys (*n* = 120)	Mean (SD) Girls (*n* = 109)	Independent *t*-Test (*t*)	*p*-Value	95% Confidence Interval
Dietary restraint T1	2.18 (0.80)	2.16 (0.75)	0.18	0.859	−0.185–0.221
Dietary restraint T2	1.95 (0.75)	1.87 (0.78)	0.76	0.446	−0.122–0.276
External eating T1	2.38 (0.88)	2.19 (0.78)	1.74	0.083	−0.025–0.409
External eating T2	2.09 (0.80)	1.92 (0.77)	1.76	0.079	−0.021–0.379
Emotional eating T1	1.83 (0.77)	1.79 (0.73)	0.42	0.673	−0.153–0.237
Emotional eating T2	1.65 (0.74)	1.59 (0.70)	0.60	0.548	−0.130–0.245

Two tailed; T1: time point 1; T2: time point 2.

**Table 2:** Descriptive statistics (means, standard deviations) and tests of difference scores (*t*) for perceived parental feeding practices for boys and girls.

Variable	Mean (SD) Boys (*n* = 120)	Mean (SD) Girls (*n* = 109)	Independent Samples *t-*Test (*t*)	*p-*Value	95% Confidence Interval
Perceived pressure to eat T1	0.98 (0.42)	0.96 (0.43)	0.25	0.806	−0.097–0.124
Perceived pressure to eat T2	0.90 (0.49)	0.85 (0.40)	0.77	0.444	−0.067–0.167
Perceived restriction T1	0.90 (0.36)	0.94 (0.35)	0.84	0.401	−0.129–0.057
Perceived restriction T2	0.90 (0.44)	0.86 (0.38)	0.87	0.385	−0.060–0.155

Two tailed; T1: time point 1; T2: time point 2.

**Table 3:** Correlation coefficients between children’s perceptions of their parents’ feeding practices at T1 and T2.

Variable	*Rs*	*p-*Value
Perception of pressure to eat	0.49	0.000
Perception of restriction	0.37	0.000

**Table 4:** Descriptive statistics and paired samples *t*-test (*t*) results for children’s perceptions of their parents’ feeding practices between T1 and T2.

Variable	T1 Mean (SD)	T2 Mean (SD)	Mean Change (SD)	Paired Samples *t*-Test (*t*)	*p*-Value	95% Confidence Interval
Perceived pressure to eat	0.97 (0.42)	0.92 (0.36)	−0.05 (0.40)	−1.80	0.070	−0.004–0.099
Perceived restriction	0.88 (0.45)	0.88 (0.41)	−0.00 (0.48)	−0.02	0.982	−0.064–0.062

Two tailed; T1: time point 1; T2: time point 2.
